# *BRCA* promoter methylation in sporadic versus *BRCA* germline mutation-related breast cancers

**DOI:** 10.1186/s13058-017-0856-z

**Published:** 2017-05-31

**Authors:** Shoko Vos, Cathy Beatrice Moelans, Paul Joannes van Diest

**Affiliations:** Department of Pathology, University Medical Center Utrecht, Heidelberglaan 100, 3584 CX Utrecht, The Netherlands

**Keywords:** Breast cancer, *BRCA*, methylation, MS-MLPA

## Abstract

**Background:**

In breast cancer, *BRCA* promoter hypermethylation and *BRCA* germline mutations are said to occur together rarely, but this property has not yet been translated into a clinical test. Our aim in this study was to investigate the diagnostic value of *BRCA1/2* methylation in distinguishing breast carcinomas of *BRCA1* and *BRCA2* germline mutation carriers from sporadic breast carcinomas using a recently developed *BRCA* methylation assay based on methylation-specific multiplex ligation-dependent probe amplification (MS-MLPA).

**Methods:**

MS-MLPAs were performed to assess *BRCA1* and *BRCA2* methylation in breast carcinoma tissues from 39 *BRCA1* and 33 *BRCA2* germline mutation carriers, 80 patients with sporadic breast cancer, and normal breast tissues from 5 *BRCA1* and 4 *BRCA2* mutation carriers and 5 nonmutation carriers.

**Results:**

Methylation frequencies varied considerably between CpG sites across the *BRCA1* and *BRCA2* promoters. Some CpG sites were methylated more frequently in *BRCA1/*2-related than in sporadic carcinomas, whereas other CpG sites were methylated more frequently in sporadic carcinomas, with large variances in sensitivity and specificity as a consequence.

**Conclusions:**

The diagnostic value of *BRCA* promoter methylation analysis in distinguishing *BRCA1/2*-related from sporadic breast carcinomas seems to be considerably dependent on the targeted CpG sites. These findings are important for adequate use of *BRCA* methylation analysis as a prescreening tool for *BRCA* germline genetic testing or to identify *BRCA*ness patients who may benefit from targeted therapies such as poly(adenosine diphosphate-ribose) polymerase inhibitors.

## Background

Breast cancer is the most frequent cancer type in women worldwide [1]. In about 5–10%, breast cancer occurs in a hereditary setting, most commonly due to *BRCA1* or *BRCA2* germline mutations, which lead to a 40–80% lifetime risk of developing breast cancer as well as a 30–40% lifetime risk of ovarian cancer development [2–8]. Promoter hypermethylation plays an important role in carcinogenesis of several organs, including the breast, because hypermethylation of cytosine phosphate guanine (CpG) sites in promoter regions may lead to downregulation of tumor suppressor genes [9–15]. It has been proposed in the literature that *BRCA* promoter hypermethylation takes place almost exclusively in the sporadic setting and only rarely occurs in patients with an underlying *BRCA1* or *BRCA2* germline mutation [16–26]. This is potentially clinically important because promoter methylation assays could then serve as prescreening tests when a hereditary nature is suspected, obviating the need for germline mutation analysis in cases of promoter methylation. However, for routine testing, more confirmation is mandatory, such as with regard to the best CpG sites to target, and a robust assay needs to be at hand that works on small amounts of fragmented DNA from formalin-fixed, paraffin-embedded (FFPE) tumor material. The latter is also important in view of the growing need to test for *BRCA1* and *BRCA2* (*BRCA1/2)* promoter methylation as a sign of *BRCA*ness, which may provide an indication for treatment with poly(adenosine diphosphate-ribose) polymerase (PARP) inhibitors [27–31]. Methylation-specific multiplex ligation-dependent probe amplification (MS-MLPA) is a rapid, robust, and inexpensive multiplex methylation test that works well on small amounts of DNA derived from FFPE tissues. The aim of this study was to investigate the diagnostic value of *BRCA1/2* promoter methylation in distinguishing breast carcinomas from *BRCA1* and *BRCA2* germline mutation carriers (*BRCA1/2*-related breast carcinomas) and sporadic breast carcinomas using a recently developed BRCA methylation MS-MLPA assay. In other words, we sought to determine to what extent *BRCA1/2* promoter methylation can be detected in *BRCA1/2*-related compared with sporadic breast carcinomas.

## Methods

### Patient material

FFPE tissues of 39 *BRCA1* and 33 *BRCA2* germline mutation-related breast cancer resection specimens (*BRCA1/2*-C) were derived from the pathology archives at the University Medical Center Utrecht, University Medical Center Groningen, VU University Medical Center Amsterdam, and local hospitals around Utrecht, The Netherlands. Also, FFPE tissues of prophylactic mastectomy specimens of five *BRCA1* and four *BRCA2* germline mutation carriers (*BRCA1/2*-N) were derived from the pathology archives of the University Medical Center Utrecht. *BRCA* status had been confirmed through mutation analysis at a medical genetics department within The Netherlands after informed consent. For comparison, FFPE tissues of 80 breast cancer resection (Sporadic-C) and 5 breast reduction samples (non-*BRCA*-related-N) from women not tested for a *BRCA* mutation were derived from the pathology archive of the University Medical Center Utrecht. These women did not receive *BRCA* germline mutation testing, because there was no clinical suspicion of a hereditary nature. No further inclusion or exclusion criteria were applied. From the tissue blocks, 4-μm-thick sections were cut and stained with hematoxylin and eosin. Tumor characterization, grading according to the modified Bloom-Richardson grading system [32], and scoring of immunohistochemical staining were performed by an experienced breast pathologist (PJvD), who was blinded to mutation status. Estrogen receptor (ER) and progesterone receptor (PR) immunohistochemical staining was considered positive when ≥10% of the tumor cells showed expression, regardless of intensity. Human epidermal growth factor receptor 2 (HER2) was scored according to the HercepTest scoring system (Dako, Glostrup, Denmark) for breast cancer, where only a 3+ score was considered positive. The clinicopathological characteristics are provided in Table 1.

**Table 1:** Clinicopathological characteristics of included breast samples

	*BRCA1*-C		*BRCA2*-C		Sporadic-C		*BRCA1*-N		*BRCA2*-N		Non-*BRCA*- related-N		*p* Value^A^
	*n*	%	*n*	%	*n*	%	*n*	%	*n*	%	*n*	%	
Number of samples (%)	39	23.5	33	19.9	80	48.2	5	3	4	2.4	5	3	
Age, years													9.56°10^−9^*ǂ
Median	43	N/A	46	N/A	58	N/A	31	N/A	36.5	N/A	22	N/A	0.549^B^
Range	30–80	N/A	21–69	N/A	29–86	N/A	29–33	N/A	35–38	N/A	18–52	N/A	
Grade^C^													0.00007*^ǂǂ^
1	0	0	1	3	20	25.3							
2	9	23.1	10	30.3	25	31.6							
3	30	76.9	22	66.7	34	43							
Tumor type													0.230^ǂǂ^
Ductal	34	87.2	29	87.9	69	86.3							
Lobular	2	5.1	2	6.1	10	12.5							
Other	3	7.7	2	6.1	1	1.3							
ER													0.0004*^ǂǂǂ^
Negative	26	66.7	8	24.2	23	28.8							
Positive	13	33.3	25	75.8	57	71.3							
PR													0.001*^ǂǂǂ^
Negative	29	74.4	17	51.5	30	37.5							
Positive	10	25.6	16	48.5	50	62.5							
HER2													0.68^ǂǂ^
Negative	38	97.4	32	97	75	93.8							
Positive	1	2.6	1	3	5	6.3							

*Abbreviations: BRCA1-C* Breast carcinomas from *BRCA1* germline mutation carriers, *BRCA2*-*C* Breast carcinomas from *BRCA2* germline mutation carriers, *BRCA1/2*-*N* Normal breast tissue from *BRCA1* and *BRCA2* germline mutation carriers, *ER* Estrogen receptor, *Non*-*BRCA*-*related*-*N* Normal breast tissue from patients not known to have a *BRCA1* or *BRCA2* germline mutation, *HER2* Human epidermal growth factor receptor 2, *N/A* Not available, *PR* Progesterone receptor, *Sporadic-C* Sporadic breast carcinoma

^A^Testing *BRCA1*-C and *BRCA2*-C together against Sporadic-C

^B^Testing *BRCA1*-N and *BRCA2*-N together against non-*BRCA*-related N

^C^For 1 of 80 sporadic breast cancer cases, the grade was unknown

^ǂ^Mann-Whitney *U* test

^ǂǂ^Fisher’s exact test

^ǂǂǂ^Pearson’s chi-square test

*Statistically significant (two-sided *p* value <0.05)

### DNA isolation

Normal breast and breast cancer tissues were harvested from 10×10-μm-thick and 4×4-μm-thick tissue sections, respectively. Areas with necrosis, preinvasive lesions, and extensive inflammation were avoided. DNA isolation was performed by overnight incubation at 56 °C in lysis buffer (50 mM Tris-HCl, pH 8.0, 0.5% Tween 20) with proteinase K (10 mg/ml; Roche, Basel, Switzerland). Proteinase K was deactivated by boiling for 10 minutes. After centrifugation for 2 minutes at 14,000 rpm, the supernatant was collected for further analysis. DNA content was measured using an ND-1000 spectrophotometer (NanoDrop Products, Wilmington, DE, USA).

### Methylation analysis

**Fig. 1:**
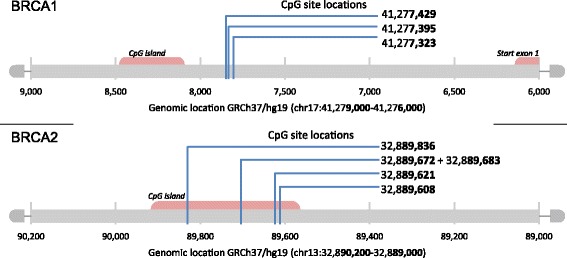
Overview of the targeted cytosine phosphate guanine (CpG) sites in the *BRCA1* and *BRCA2* promoter regions by methylation analysis of the selected studies

Five microliters of supernatant with a DNA concentration between 50 and 500 ng/μl were used for MS-MLPA analysis according to the manufacturer’s instructions, using the ME053 *BRCA1*-*BRCA2* X1-0914 methylation assay (MRC-Holland, Amsterdam, The Netherlands). When the DNA concentration exceeded 500 ng/μl, the input volume was adjusted proportionally. The ME053 methylation assay contains three and four probes to detect *BRCA1* and *BRCA2* promoter methylation, respectively, enabling methylation status determination of three CpG sites in the *BRCA1* promoter region and five CpG sites in the *BRCA2* promoter region (*see* Table 2 and Fig. 1 for further details). The MS-MLPA principle and analytical procedure are described elsewhere [33], and the technique has been shown to be reliable for methylation assessment [33–37]. Samples that were 100% methylated (SssI methyltransferase-treated MDA-MB-231 and A549 cells) were used as positive controls, and normal peripheral blood samples were used as negative controls. No template controls were included. Moreover, the methylation assay included two digestion (methylation) control probes.

**Table 2:** Methylation probe characteristics of the methylation-specific multiplex ligation-dependent probe amplification assay (ME053 *BRCA1*-*BRCA2* X1-0914; MRC-Holland)

Gene	Probe ID	Length	Chromosome position	5′ Probe	3′ Probe	Start	End	CpG site	CpG loci ID
*BRCA1*	BRCA1.1	165	17q21.31	CCTCTGAGAGGCTGCTGCTTAGCGGTAGCCCCTT	GGTTTCCGTGGCAACGGAAAAGCGCGGGAATTACAGA	41277415	41277483	41277429	cg04110421
*BRCA1*	BRCA1.2	230	17q21.31	CATGCATCTGAGAAACCCCACAGCCTGTCCCCCGTCCAGGAA	GTCTCAGCGAGCTCACGCCGCGCAGTCGCAGTTT	41277407	41321886	41277395	cg16630982
*BRCA1*	BRCA1.3	252	17q21.31	GTGGGGTTTCTCAGATAACTGGGCCCCTGC	GCTCAGGAGGCCTTCACCCTCTGCTCTGGGTAAAGGT	41277286	41277352	41277323	cg08993267
*BRCA2*	BRCA2.1	130	13q13.1	CCATCTTGTGGCGCGAGCTTCTGAAACTA	GGCGGCAGAGGCGGAGCCGCTGTGGCACTGCT	32889614	32889670	32889621	N/A
*BRCA2*	BRCA2.2	149	13q13.1	TGCGGGTTAGTGGTGGTGGTAGTGGGTT	GGGACGAGCGCGTCTTCCGCAGTCCCAGTCCAGCGTGG	32889801	32889865	32889836	N/A
*BRCA2*	BRCA2.3	160	13q13.1	CCTCTGAGAGGCTGCTGCTTAGCGGTAGCCCCTT	GGTTTCCGTGGCAACGGAAAAGCGCGGGAATTACAGA	32889665	32889746	32889672 + 32889683	N/A
*BRCA2*	BRCA2.4	217	13q13.1	CTTCCGGGTGGTGCGTGTGCTGCGTGTCGC	GTCACGGCGTCACGTGGCCAGCGCGGGCTTGT	32889557	32889618	2889608	N/A

*N/A* Not available

The chromosomal locations are based upon GRCh37/hg19. The first nucleotides of the 5′ probe may not be complementary to the target DNA, because it contains the stuffer sequence. The CpG loci ID are as determined by the Infinium HumanMethylation450 BeadChip Array (Illumina, San Diego, CA, USA)

**Fig. 2:**
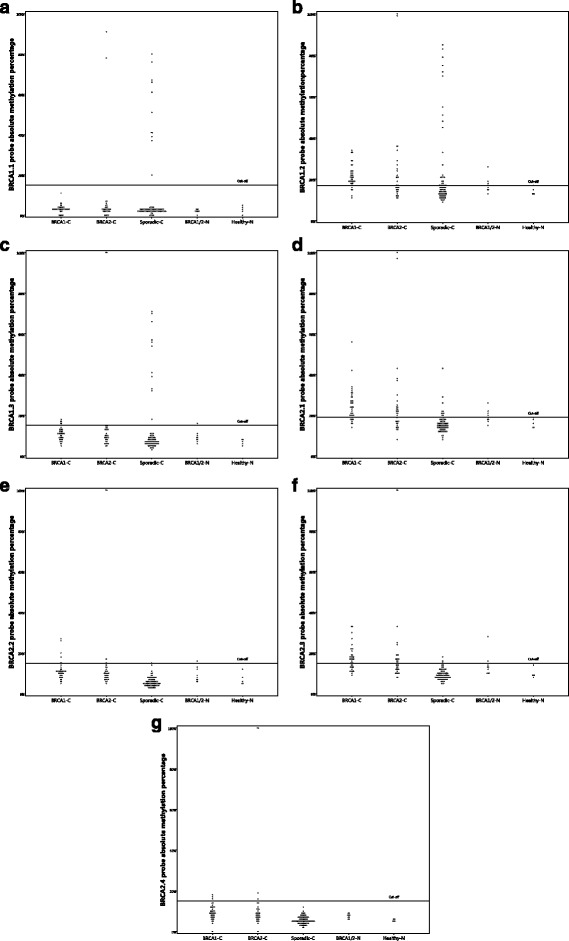
**a**–**g** Distribution of absolute methylation percentages for all *BRCA1* and *BRCA2* methylation probes. *BRCA1*-*C* Breast carcinomas from *BRCA1* germline mutation carriers, *BRCA2*-*C* Breast carcinomas from *BRCA2* germline mutation carriers, *BRCA1/2*-*N* Normal breast tissue from *BRCA1* and *BRCA2* germline mutation carriers, *Sporadic-C* Sporadic breast carcinoma

Coffalyser.Net software (MRC-Holland) was used for methylation data analysis. Quality control showed that the results of the control probes and control samples were adequate. The methylation percentage cutoff per probe was set at the highest methylation percentage value in normal breast tissues from nonmutation carriers (non-*BRCA*-related N), ranging from >15% to >19% (*see also* Fig. 2). Moreover, the cumulative methylation index (CMI) was calculated as the sum of the methylation percentage of all methylation probes. MS-MLPA analysis was performed by SV and CBM, who were blinded to mutation status.

### Statistical analysis

Statistical analyses were performed using IBM SPSS Statistics version 23.0 software (IBM, Armonk, NY, USA). Associations between absolute methylation percentages, CMI or age, and mutation status (*BRCA1/2*-related carcinomas versus sporadic carcinomas) were assessed by the Mann-Whitney *U* test. Associations between dichotomized *BRCA* promoter methylation and mutation status or other clinicopathological characteristics were assessed by Pearson’s chi-square test or Fisher’s exact test. Sensitivity and specificity were calculated. Correlations between CMI and age were assessed using the Spearman’s rho correlation coefficient. The level of significance used was set at a two-sided *p* value <0.05.

### Correlation between *BRCA1/2* methylation and messenger RNA expression

The Wanderer tool was used to assess the correlation between *BRCA1/2* methylation and messenger RNA (mRNA) expression. This tool was created on the basis of data from The Cancer Genome Atlas (TCGA) Research Network [38]. The Infinium 450K HumanMethylation Array (Illumina, San Diego, CA, USA) was selected as the methylation data type, and Spearman’s correlation coefficient was selected as the correlation method.

## Results

### *BRCA1* promoter methylation in *BRCA1/2*-related and sporadic breast carcinomas

The absolute methylation percentages and their distribution varied considerably between the three *BRCA1* methylation probes (Table 3, Fig. 2). For the BRCA1.2 and BRCA1.3 probes, *BRCA1/*2-C showed significantly higher median methylation percentages than Sporadic-C (*p* = 0.00006 and *p* = 0.00003, respectively). The dichotomized results are shown in Table 4. *BRCA1/2*-C showed significantly less frequent methylation with the BRCA1.1 probe (*p* = 0.019), but significantly more frequent methylation with the BRCA1.2 probe (*p* = 0.000009). Methylation of at least one of the three *BRCA1* methylation probes was seen in 46 (63.9%) of 72 *BRCA1/2*-C compared with 22 (27.5%) of 80 Sporadic-C (*p* = 0.000009). The sensitivity and specificity of the *BRCA1* methylation probes in distinguishing *BRCA1/2*-C from Sporadic-C are shown in Table 5. The calculation of the sensitivity and specificity differs between probes owing to differences in methylation frequencies between *BRCA1/2*-C and Sporadic-C (*see* Table 4 and explanation in Table 5). The BRCA1.1 and BRCA1.3 probes showed good performance in ruling out *BRCA1/2* germline mutations when methylation was detected (sensitivity 97.2% and 90.3%, respectively), although the specificity was poor because many Sporadic-C did not show methylation with these probes either (specificity for both 13.8%). The BRCA1.2 probe and the combination of the three *BRCA1* probes (*BRCA1* total) showed moderate sensitivity (both 63.9%) and specificity (72.5%) when used to rule in *BRCA1/2* germline mutations when methylation was present.

**Table 3:** *BRCA* promoter methylation percentages in *BRCA1/2*-related and sporadic breast carcinomas by methylation-specific multiplex ligation-dependent probe amplification

Probe	*BRCA1*C-C, median % (range)	*BRCA2*-C, median % (range)	Sporadic-C, median % (range)	Test statistic^a^	*p* Value
BRCA1.1	3 (0–11)	3 (0–91)	3 (0–80)	2494.500	0.140
BRCA1.2	21 (11–34)	17 (11–100)	15 (9–85)	1795.500	0.00006*
BRCA1.3	11 (5–18)	9 (5–100)	7 (3–71)	1760.600	0.00003*
BRCA2.1	24 (14–56)	21 (8–100)	15 (8–43)	947.500	9.85°10^−13^*
BRCA2.2	11 (5–27)	10 (5–100)	5 (3–15)	596.000	1.00°10^−13^*
BRCA2.3	17 (9–33)	14 (8–100)	9 (5–18)	536.500	1.00°10^−13^*
BRCA2.4	9 (0–18)	8 (0–100)	5 (2–12)	984.500	1.95°10^−12^*

*Abbreviations: BRCA1-C* Breast carcinomas from *BRCA1* germline mutation carriers, *BRCA2-C* Breast carcinomas from *BRCA2* germline mutation carriers, *Sporadic-C* Sporadic breast carcinoma

^a^Mann-Whitney *U* test for *BRCA1*-C and *BRCA2*-C together against Sporadic-C

*Statistically significant (two-sided *p* value <0.05)

**Table 4:** Frequency of *BRCA* methylation (dichotomized results) in *BRCA1/2*-related and sporadic breast carcinomas by methylation-specific multiplex ligation-dependent probe amplification

Probe	*BRCA1*-C, total *n* = 39 (%)	*BRCA2*-C, total *n* = 33 (%)	Sporadic-C, total *n* = 80 (%)	Test statistic^a^	*P*-value	Total (%)	Cutoff^b^
BRCA1.1	0 (0)	2 (6.1)	11 (13*.*8)	5.833^c^	0*.*019*	13 (8*.*6)	>15%
BRCA1.2	32 (82.1)	14 (42.4)	22 (27*.*5)	20.296^c^	0*.*000009*	68 (44.7)	>17%
BRCA1.3	5 (12.8)	2 (6.1)	11 (13*.*8)	0.589^c^	0*.*465	18 (11.8)	>15%
*BRCA1* total^d^	32 (82.1)	14 (42.4)	22 (27.5)	20.296^c^	0.000009*	68 (44.7)	
BRCA2.1	30 (76.9)	18 (54.5)	10 (12*.*5)	47.117^c^	2.93°10^−12^*	58 (38.2)	>19%
BRCA2.2	4 (10*.*3)	3 (9.1)	0 (0)	8.153^e^	0*.*005*	7 (4*.*6)	>15%
BRCA2.3	22 (56.4)	14 (42.4)	2 (2*.*5)	45.600^c^	1.65°10^−12^*	38 (25)	>15%
BRCA2.4	3 (7*.*7)	4 (12.1)	0 (0*.*0)	8.153^e^	0*.*005*	7 (4*.*6)	>15%
*BRCA2* total^d^	30 (76.9)	20 (60.6)	10 (12.5)	51.432^c^	0.029*	60 (39.5)	

*Abbreviations: BRCA1-C* Breast carcinomas from *BRCA1* germline mutation carriers, *BRCA2-C* Breast carcinomas from *BRCA2* germline mutation carriers, *Sporadic-C* Sporadic breast carcinoma

^a^Pearson’s chi-square test or Fisher’s exact test for *BRCA1*-C and *BRCA2*-C together against Sporadic-C

^b^Cutoff based upon highest methylation percentage detected in normal breast tissue from nonmutation carriers

^c^Pearson’s chi-square test

^d^ *BRCA1*-C and *BRCA2*-C total entails the number (and percentage) of samples showing methylation in at least one of the *BRCA1* or *BRCA2* probes, respectively

^e^Fisher’s exact test

*Statistically significant (two-sided *p* value <0.05)

**Table 5:** Sensitivity and specificity for each methylation probe in distinguishing *BRCA1/2*-related from sporadic breast carcinomas

Probe	Sensitivity	95% CI	Specificity	95% CI
BRCA1.1^a^	70 of 72 (97.2%)	90.3–99.6%	11 of 80 (13.8%)	7.1–23.3%
BRCA1.2^b^	46 of 72 (63.9%)	51.7–74.9%	58 of 80 (72.5%)	61.4–81.9%
BRCA1.3^a^	65 of 72 (90.3%)	90.0–96.0%	11 of 80 (13.8%)	7.1–23.3%
*BRCA1* total^c^	*46 of 72 (63.9%)*	*51.7–74.9%*	*58 of 80 (72.5%)*	*61.4–81.9%*
BRCA2.1^b^	48 of 72 (66.7%	54.6–77.3%	70 of 80 (87.5%)	78.2–93.8%
BRCA2.2^b^	7 of 72 (9.7%)	4.0–19.0%	80 of 80 (100%)	95.5–100%
BRCA2.3^b^	36 of 72 (50.0%)	38.0–62.0%	78 of 80 (97.5%)	91.3–99.7%
BRCA2.4^b^	7 of 72 (9.7%)	4.0–19.0%	80 of 80 (100%)	95.5–100%
*BRCA2* total^c^	*50 of 72 (69.4%)*	*57.5–79.8%*	*70 of 80 (87.5%)*	*78.2–93.8%*

^a^Sensitivity and specificity calculated as if *BRCA1* promoter methylation would be performed to rule out *BRCA* germline mutations. True-positive: *BRCA1/2*-related cancers without BRCA1.1 or BRCA1.3 methylation. True-negative: sporadic cancers with BRCA1.1 or BRCA1.3 methylation

^b^Sensitivity and specificity calculated as if *BRCA1* promoter methylation would be performed to rule in *BRCA* germline mutations. True-positive: *BRCA1/2*-related cancers with BRCA1.2, BRCA2.1, BRCA2.2, BRCA2.3, or BRCA2.4 methylation. True-negative: sporadic cancers without BRCA1.2, BRCA2.1, BRCA2.2, BRCA2.3, or BRCA2.4 methylation

^c^Sensitivity and specificity calculated as if *BRCA1* promoter methylation would be performed to rule in *BRCA* germline mutations. True-positive: *BRCA1/2*-related cancers with methylation of at least one of the *BRCA1* or *BRCA2* probes. True-negative: sporadic cancers without methylation in any of the *BRCA1* or *BRCA2* probes

To evaluate the robustness of the MS-MLPA assay, we compared the results of the ME053 assay with another MS-MLPA assay tested on Sporadic-C in our laboratory, the ME001 assay (MRC-Holland) (C. B. Moelans, unpublished observations; data not shown). One of the *BRCA1* methylation probes in this assay determines the methylation status of the same CpG site as the BRCA1.3 probe in the ME053 MS-MLPA assay. There was a strong correlation in dichotomized *BRCA1* promoter methylation results in Sporadic-C between the two assays (Spearman’s rho correlation coefficient, 0.831; *p* = 1000°10^−13^). For absolute methylation percentages, the correlation was weaker but still significant (Spearman’s rho correlation coefficient, 0.379; *p* = 0.001). In general, the Sporadic-C showed slightly higher *BRCA1* methylation percentages with the ME001 assay. In 4 of 80 cases, *BRCA1* was methylated according to the ME001 assay but unmethylated according to the ME053 assay. However, methylation percentages in these cases were only slightly above the threshold of 15% (17–20%) with the ME001 assay.

### *BRCA2* promoter methylation in *BRCA1/2*-related and sporadic breast carcinomas

*BRCA1/2*-C showed significantly higher median methylation percentages for all *BRCA2* methylation probes than Sporadic-C, although the absolute methylation percentages and their distribution varied considerably between the four *BRCA2* methylation probes (Table 3, Fig. 2). Using dichotomized results, *BRCA1/2*-C showed significantly more frequent methylation in all four probes, as shown in Table 4. When the dichotomized results of the *BRCA2* methylation probes were combined, 50 (69.4%) of 72 *BRCA1/2*-C showed methylation of at least one of the four *BRCA2* methylation probes, compared with 10 (12.5%) of 80 Sporadic-C (*p* = 0.029). The sensitivity and specificity of the *BRCA1* methylation probes in distinguishing *BRCA1/2*-C from Sporadic-C are shown in Table 5. The BRCA2.2 and BRCA2.4 probes showed excellent specificity (both 100%) when used to rule in *BRCA1/2* germline mutations when methylation was detected because no Sporadic-C were methylated with these probes. However, the sensitivity was poor (both 9.7%) because few *BRCA1/2*-C showed methylation. The BRCA2.1 and BRCA2.3, as well as the combination of all four *BRCA2* probes (*BRCA2* total), showed moderate sensitivity (50.0–69.4%) and rather good specificity (87.5–97.5%) when used to rule in *BRCA1/2* germline mutations when methylation was detected.

### Correlation with clinicopathological variables

As shown in Table 1, *BRCA1/2*-C and Sporadic-C differed significantly with respect to age, grade, and ER and PR status. We analyzed whether the differences we observed in methylation frequencies between *BRCA1/2*-C and Sporadic-C may be related to these differences in clinicopathological variables (Tables 6, 7 and 8). In Sporadic-C, methylation of the BRCA1.1, BRCA1.2, and BRCA1.3 probes, separately as well as combined (*BRCA1* total), was significantly more frequently detected in grade 3 tumors than in grades 1–2 tumors and in ER-negative than in ER-positive tumors. Methylation of the BRCA1.1 and BRCA1.3 probes was also significantly more frequently detected in PR-negative tumors. For *BRCA2* methylation in Sporadic-C, there was a statistically significant association only with grade: Methylation of the BRCA2.1 probe and of all four *BRCA2* probes combined was more frequently seen in grade 3 carcinomas. There were no statistically significant correlations between *BRCA1* and *BRCA2* methylation on the one hand and between tumor type (ductal versus lobular carcinomas) and HER2 status on the other hand. In *BRCA1*-C and *BRCA2*-C, there were no statistically significant associations between *BRCA1* or *BRCA2* methylation and clinicopathological variables. Moreover, no statistically significant correlation was found between CMI for *BRCA1* and/or *BRCA2* promoter methylation and age in *BRCA1*-C, *BRCA2*-C, Sporadic-C, *BRCA1/2*-N, and non-*BRCA*-related-N (Table 9).

**Table 6:** Relationship between *BRCA1/2* methylation and clinicopathological variables in sporadic breast carcinomas

Sporadic carcinomas	Grades 1–2 vs. 3		Ductal vs. lobular tumors		ER-positive vs. ER-negative		PR-positive vs. PR-negative		HER2-positive vs. HER2-negative	
Probe	Test statistic^a^	*p* Value	Test statistic^a^	*p* Value	Test statistic^a^	*p* Value	Test statistic^a^	*p* Value	Test statistic^a^	*p* Value
BRCA1.1	12.230^b^	0.001*	1.852^b^	0.342	7.577^b^	0.011*	6.753^b^	0.016*	0.176^b^	0.533
BRCA1.2	8.190^c^	0.006*	0.026^b^	1.000	6.689^c^	0.014*	3.762^c^	0.071	2.825^b^	0.125
BRCA1.3	12.230^b^	0.001*	1.852^b^	0.342	7.577^b^	0.011*	6.753^b^	0.016*	0.176^b^	0.533
*BRCA1* total^c^	8.190^c^	0.006*	0.026^b^	1.000	6.689^c^	0.014*	3.762^c^	0.071	2.825^b^	0.125
BRCA2.1	6.577^b^	0.015*	1.659^b^	0.345	0.706^b^	0.462	0.274^b^	0.736	3.688^b^	0.115
BRCA2.2	N/A	N/A	N/A	N/A	N/A	N/A	N/A	N/A	N/A	N/A
BRCA2.3	2.775^b^	0.178	0.297^b^	1.000	5.084^b^	0.080	0.137^b^	1.000	0.137^b^	1.000
BRCA2.4	N/A	N/A	N/A	N/A	N/A	N/A	N/A	N/A	N/A	N/A
*BRCA2* total^c^	6.577^b^	0.015*	1.659^c^	0.345	0.706^c^	0.462	0.274^c^	0.736	3.688^c^	0.115

*Abbreviations: ER* Estrogen receptor, *HER2* Human epidermal growth factor receptor 2, N/A Not applicable, because one of the two variables (either the methylation probe or the clinicopathological variable) was a constant, *PR* Progesterone receptor

^a^Pearson’s chi-square test or Fisher’s exact test

^b^Fisher’s exact test

^c^Pearson’s chi-square test

*Statistically significant (two-sided *p* value <0.05)

**Table 7:** Relationship between *BRCA1/2* methylation and clinicopathological variables in *BRCA1*-related carcinomas

*BRCA1*-related carcinomas	Grades 1–2 vs. 3		Ductal vs. lobular tumors		ER-positive vs. ER-negative		PR-positive vs. PR-negative		HER2-positive vs. HER2-negative	
Probe	Test statistic^a^	*p* Value	Test statistic^a^	*p* Value	Test statistic^a^	*p* Value	Test statistic^a^	*p* Value	Test statistic^a^	*p* Value
BRCA1.1	N/A	N/A	N/A	N/A	N/A	N/A	N/A	N/A	N/A	N/A
BRCA1.2	0.145^b^	0.653	1.262^b^	0.356	0.087^b^	1.000	0.038^b^	1.000	0.225^b^	1.000
BRCA1.3	0.031^b^	1.000	0.342^b^	1.000	0.115^b^	1.000	0.096^b^	1.000	0.151^b^	1.000
*BRCA1* total^c^	0.145^b^	0.653	1.262^b^	0.356	0.087^b^	1.000	0.038^b^	1.000	0.225^b^	1.000
BRCA2.1	0.005^b^	1.000	1.694^b^	0.310	0.000^b^	1.000	0.363^b^	0.669	0.308^b^	1.000
BRCA2.2	0.009^b^	1.000	0.265^b^	1.000	3.482^b^	0.099	0.001^b^	1.000	0.117^b^	1.000
BRCA2.3	0.003^b^	1.000	3.328^b^	0.144	0.209^bb^	0.740	0.070^b^	1.000	0.793^b^	1.000
BRCA2.4	0.975^b^	1.000	0.193^b^	1.000	1.625^b^	0.253	1.121^b^	0.556	0.086^b^	1.000
*BRCA2* total^c^	0.005^b^	1.000	1.694^b^	0.310	0.000^b^	1.000	0.363^b^	0.669	0.308^b^	1.000

*Abbreviations: BRCA1/2 BRCA1* and *BRCA2*, *ER* Estrogen receptor, *HER2* Human epidermal growth factor receptor 2, *N/A* Not applicable, because one of the two variables (either the methylation probe or the clinicopathological variable) was a constant, *PR* Progesterone receptor

^a^Pearson’s chi-square test or Fisher’s exact test

^b^Fisher’s exact test

^c^Pearson’s chi-square test

**Table 8:** Relationship between *BRCA1/2* methylation and clinicopathological variables in *BRCA2*-related carcinomas

*BRCA2*-related carcinomas	Grades 1–2 vs. 3		Ductal vs. lobular tumors		ER-positive vs. ER-negative		PR-positive vs. PR-negative		HER2-positive vs. HER2-negative	
Probe	Test statistic^a^	*p* Value	Test statistic^a^	*p* Value	Test statistic^a^	*p* Value	Test statistic^a^	*p* Value	Test statistic^a^	*p* Value
BRCA1.1	1.065^b^	0.542	0.147^b^	1.000	0.769^b^	0.432	0.002^b^	1.000	0.067^b^	1.000
BRCA1.2	0.062^b^	1.000	0.057^b^	1.000	0.248^b^	0.695	0.730^c^	0.491	0.760^b^	1.000
BRCA1.3	1.065^b^	0.542	0.147^b^	1.000	0.769^b^	0.432	0.002^b^	1.000	0.067^b^	1.000
*BRCA1* total^c^	0.062^b^	1.000	0.057^b^	1.000	0.248^b^	0.695	0.730^bb^	0.491	0.760^b^	1.000
BRCA2.1	2.200^c^	0.266	0.002^b^	1.000	0.088^b^	1.000	2.528^c^	0.166	1.238^b^	0.455
BRCA2.2	1.650^b^	0.534	0.229^b^	1.000	0.149^b^	1.000	0.437^b^	0.601	0.103^b^	1.000
BRCA2.3	3.039^b^	0.136	0.115^b^	1.000	1.742^b^	0.238	0.022^c^	1.000	0.760^b^	1.000
BRCA2.4	0.569^b^	0.586	0.317^b^	1.000	0.001^b^	1.000	0.004^b^	1.000	0.142^b^	1.000
*BRCA2* total^c^	3.110^b^	0.132	0.057^b^	1.000	0.916^b^	0.431	0.863^c^	0.481	1.587^b^	0.394

*Abbreviations: BRCA1/2 BRCA1* and *BRCA2*, *ER* Estrogen receptor, *ER* Estrogen receptor, *HER2* Human epidermal growth factor receptor 2, *PR* Progesterone receptor

^a^Pearson’s chi-square test or Fisher’s exact test

^c^Pearson’s chi-square test

^b^Fisher’s exact test

**Table 9:** Correlation between age and cumulative methylation index for *BRCA1/2* methylation

Age	CMI for *BRCA1*		CMI for *BRCA2*		CMI for *BRCA1* + *BRCA2*	
	Spearman’s rho	*p* Value	Spearman’s rho	*p* Value	Spearman’s rho	*p* Value
*BRCA1*-C	−0.297	0.066	−0.287	0.077	−0.275	0.090
*BRCA2*-C	−0.019	0.918	0.003	0.989	−0.035	0.846
Sporadic-C	−0.153	0.175	0.003	0.982	−0.120	0.289
*BRCA1/*2-N	−0.252	0.513	−0.467	0.205	−0.417	0.265
Non-*BRCA*-related-N	0.300	0.624	0.100	0.873	0.100	0.873

*Abbreviations: BRCA1/2 BRCA1* and *BRCA2*, *BRCA1*-C Breast carcinomas from *BRCA1* germline mutation carriers, *BRCA2*-C Breast carcinomas from *BRCA2* germline mutation carriers, *BRCA1/2*-N Normal breast tissue from *BRCA1* and *BRCA2* germline mutation carriers, CMI Cumulative methylation index, Non-*BRCA*-related-N Normal breast tissue from patients not known to have a *BRCA1* or *BRCA2* germline mutation, Sporadic-C Sporadic breast carcinoma

Correlation between age and CMI measured by Spearman’s rho correlation coefficient. CMI is calculated as the sum of the methylation percentage of all *BRCA1* or *BRCA2* methylation probes

### Correlation between *BRCA1/2* methylation and mRNA expression

Methylation of the evaluated CpG sites within the *BRCA1* and *BRCA2* promoters showed weak correlations with mRNA levels by TCGA data extraction through the Wanderer viewer. The Spearman’s correlation coefficients between *BRCA1* methylation and mRNA expression were −0.203 for cg04110421 (targeted by the BRCA1.1 probe), −0.296 for cg16630982 (targeted by the BRCA1.2 probe), and −0.172 for cg08993267 (targeted by the BRCA1.3 probe). For *BRCA2*, the CpG loci identifiers from the TCGA data most closely located to our MS-MLPA targets were used. Therefore, the correlation between *BRCA2* methylation and mRNA expression should be interpreted with caution. The Spearman’s correlation coefficients between *BRCA2* methylation and mRNA expression were −0.014 for cg20073910 (82 and 69 bp from CpG sites targeted by the BRCA2.1 and BRCA2.4 probes, respectively), 0.067 for cg27253386 (80 and 69 bp from the CpG sites targeted by the BRCA2.3 probe), and −0.092 for cg08157964 (25 bp from the CpG site targeted by the BRCA2.2 probe).

### *BRCA* promoter methylation in *BRCA1/2*-related and non-*BRCA*-related normal breast tissue

*BRCA1/2*-N samples showed statistically significant higher absolute methylation percentages for the BRCA2.3 and BRCA2.4 probes (*p* = 0.031 and *p* = 0.005, respectively) (Table 10, Fig. 2). There was a borderline significant trend of higher methylation percentages for the BRCA1.2, BRCA1.3, and BRCA2.1 probes in *BRCA1/2*-N samples than for the non-*BRCA*-related-N cases (Table 10, Fig. 2). If methylation cutoffs per probe were based upon the highest methylation percentage found in non-*BRCA*-related-N cases, 40% (two of five) and 60% (three of five) of *BRCA1*-N cases would have at least one methylated *BRCA1* probe and one methylated *BRCA2* probe, respectively. *BRCA2*-N cases would have methylation of at least one *BRCA1* probe and one *BRCA2* probe in 25% (one of four) and 50% (two of four) of cases, respectively (Table 11).

**Table 10:** *BRCA* promoter methylation percentages in normal breast from *BRCA1/2* germline mutation carriers and nonmutation carriers

Probe	*BRCA1/2*-N, median % (range)	Non-*BRCA*-related-N, median % (range)	Test statistic^a^	*p* Value
BRCA1.1	2 (0–3)	3 (0–5)	15.500	0.325
BRCA1.2	17 (13–26)	13 (13–17)	8.500	0.057
BRCA1.3	9 (6–16)	7 (5–8)	8.000	0.050
BRCA2.1	19 (15–26)	16 (14–19)	8.500	0.060
BRCA2.2	8 (6–16)	6 (5–12)	12.000	0.158
BRCA2.3	13 (10–28)	9 (8–14)	6.500	0.031*
BRCA2.4	8 (6–9)	5 (5–6)	2.000	0.005*

*Abbreviations: BRCA1/2*-N, Normal breast tissue from *BRCA1* and *BRCA2* germline mutation carriers; Non-*BRCA*-related-N, Normal breast tissue from patients not known to have a *BRCA1* or *BRCA2* germline mutation

^a^Mann-Whitney *U* test for *BRCA1/*2-N together against non-*BRCA*-related-N

*Statistically significant (two-sided *p* value <0.05)

**Table 11:** Frequency of *BRCA* methylation (dichotomized) in prophylactic mastectomies of *BRCA1/2* germline mutation carriers by methylation-specific multiplex ligation-dependent probe amplification

Probe	*BRCA1*, total *n* = 5 (%)	*BRCA2*, total *n* = 4 (%)	Total, *n* = 9 (%)	Cutoff^a^
BRCA1.1	0 (0)	0 (0)	0 (0)	>15%
BRCA1.2	2 (40)	1 (25)	3 (30)	>17%
BRCA1.3	1 (20)	0 (0)	1 (11.1)	>15%
*BRCA1* total^b^	2 (40)	1 (25)	3 (30)	
BRCA2.1	2 (40)	2 (50)	4 (44.4)	>19%
BRCA2.2	1 (20)	0 (0)	1 (11.1)	>15%
BRCA2.3	2 (50)	0 (0)	2 (22.2)	>15%
BRCA2.4	0 (0)	0 (0)	0 (0)	>15%
*BRCA2* total^b^	3 (60)	2 (50)	5 (55.5)	

^a^Cutoff based upon highest methylation percentage detected in normal breast tissue from nonmutation carriers

^b^ *BRCA1* and *BRCA2* total entails the number and percentage of samples showing methylation in at least one of the *BRCA1* or *BRCA2* probes, respectively

## Discussion

The aim of this study was to investigate the diagnostic value of *BRCA1/2* promoter methylation analysis using a new *BRCA* methylation MS-MLPA assay in distinguishing sporadic breast carcinomas from *BRCA1* and *BRCA2* germline mutation-related carcinomas in order to arrive at a clinically applicable prescreening test for *BRCA1/2*-related cancers. We observed considerably varying frequencies of *BRCA* promoter methylation between the targeted CpG sites across the *BRCA1* and *BRCA2* promoters. Some CpG sites were methylated more frequently in *BRCA1/*2-C than in Sporadic-C (those targeted by the BRCA1.2, BRCA2.1, BRCA2.2, BRCA2.3, and BRCA2.4 probes), whereas other CpG sites were methylated more frequently in Sporadic-C (those targeted by the BRCA1.1 and BRCA1.3 probes). In general, we observed frequent *BRCA* promoter methylation in *BRCA1/2*-C. At least 63.8% (46 of 72) of *BRCA1/2*-C and 12.5% (10 of 80) of Sporadic-C showed methylation of at least one of the targeted CpG sites in the *BRCA1* or *BRCA2* promoter. Interestingly, several *BRCA1*-C showed *BRCA2* promoter methylation and vice versa. Sensitivity and specificity varied considerably between the probes. The best probes for ruling out Sporadic-C when methylation was detected were BRCA2.2 and BRCA2.4 (specificity 100%). However, many *BRCA1/2*-C would be missed because the sensitivity was poor (9.7%). The best probes for ruling out *BRCA1/2*-C when methylation was not detected were BRCA1.1 and BRCA1.3 (sensitivity 97.2% and 90.3%, respectively). However, many Sporadic-C would be misclassified as potentially *BRCA1/2* germline mutation-related because the specificity was poor (both 13.8%). Sensitivity and specificity were most balanced when all four *BRCA2* probes were used to rule in *BRCA1/2* germline mutations when methylation was detected in at least one the *BRCA2* probes (sensitivity 69.4%, specificity 87.5%). *BRCA1* promoter methylation was more frequent in high-grade, ER-negative, and PR-negative tumors. This finding is in line with other reports in the literature because *BRCA1* methylation has been more frequently described in triple-negative breast carcinomas [39, 40]. *BRCA2* promoter methylation was more frequent in high-grade tumors but showed no other statistically significant clinicopathological associations.

In line with our findings, Daniels et al. [41] recently demonstrated that DNA methylation levels vary between CpG sites in the *BRCA1* promoter. However, our findings do not support the general assumption and previous findings reported in the literature that *BRCA* promoter methylation and *BRCA* germline mutations are mutually exclusive. In most studies, none of the *BRCA*-related breast carcinomas showed *BRCA* promoter methylation [16, 21, 23–26]. Kontorovich et al. [20] observed *BRCA1* promoter methylation in 3 (6.3%) of 48 *BRCA1*-related breast carcinomas, and Tapia et al. [17] observed *BRCA1* promoter methylation in 2 (66.7%) of 3 observed *BRCA1*-related breast carcinomas. Differences in observed methylation frequencies could be related to the technique and specific CpG sites targeted, the quality of input material, and the determination of methylation cutoffs in subsequent analysis. It should be noted that some patients with a *BRCA* germline mutation may develop breast cancer through sporadic breast carcinogenetic mechanisms, which could affect methylation frequencies.

Whether *BRCA* promoter methylation may occur as a second hit in *BRCA1/2*-related breast carcinomas is still unclear. The main question is whether methylation really drives carcinogenesis or whether it can be considered a bystander. Interestingly, in our study, normal breast tissues from *BRCA1/2* germline mutation carriers showed *BRCA2* promoter methylation levels compared with normal breast tissues from patients without *BRCA* germline mutations, although the sample size was limited. Bijron et al. [42] described increased *BRCA2* promoter methylation in normal and precursor fallopian tube tissues from *BRCA* germline mutation carriers compared with normal sporadic fallopian tube tissues. *BRCA* methylation might therefore play a role in carcinogenesis in a subset of *BRCA* germline mutation carriers.

To our knowledge, our present study is the largest one to date investigating both *BRCA1* and *BRCA2* promoter methylation in *BRCA1* as well as *BRCA2* germline mutation-related breast carcinomas. Moreover, this is the first MS-MLPA study to specifically test *BRCA* promoter methylation in *BRCA1*- and *BRCA2*-related breast carcinomas compared with sporadic breast carcinomas, as well as the first MS-MLPA study in which *BRCA* methylation levels have been investigated in normal breast tissues of *BRCA* carriers. We validated our results for one of the BRCA methylation probes by comparing them with data obtained from a previous MS-MLPA experiment using the commercially available ME001 MS-MLPA assay.

Our findings may have important implications for clinical practice, such as prescreening for *BRCA* germline genetic testing or eligibility for certain therapeutic strategies. *BRCA1* promoter methylation analysis has been proposed as a cost-effective and reliable prescreening tool to exclude *BRCA1* germline mutations in patients with breast cancer similar to *MLH1* promoter methylation and Lynch syndrome [22, 43]. Moreover, recent research shows that breast and ovarian carcinomas with *BRCA* deficiencies, including *BRCA* methylation, may also benefit from PARP inhibitor therapy [30, 31, 44–49].

Although MS-MLPA has been shown to be a reliable tool to assess methylation in general, it targets single specific sites targetable by the HhaI methylation-sensitive restriction enzyme. For MS-MLPA to be a reliable prescreening tool for ruling in or ruling out *BRCA* germline mutations and/or determining sensitivity for targeted therapy, a review of existing literature and further research, preferably assessing all CpG sites in the *BRCA* promoter regions (e.g., by methylation-specific polymerase chain reaction), is needed to determine the most predictive CpG sites for each indication. The most predictive CpG sites should then be targetable by the HhaI methylation-sensitive restriction enzyme because otherwise MS-MLPA may not be the preferred methylation analytical technique in this context.

## Conclusions

The diagnostic value of *BRCA* promoter methylation analysis in distinguishing *BRCA1/2*-related and sporadic breast carcinomas is considerably dependent on the targeted CpG sites. These findings are important for adequate use of *BRCA* methylation analysis as a prescreening tool for germline genetic testing or to identify patients who may benefit from targeted therapies such as PARP inhibitors, making their way to the clinic for breast cancer. Further research is needed to assess which other CpG sites are important in ruling in or ruling out *BRCA* germline mutations or determining sensitivity for targeted therapy.

## Abbreviations

*BRCA1/2*: *BRCA1* and *BRCA2*
*BRCA1*-C: Breast carcinomas from *BRCA1* germline mutation carriers
*BRCA2*-C: Breast carcinomas from *BRCA2* germline mutation carriers
*BRCA1/2*-related: Related to a *BRCA1* or *BRCA2* germline mutation
*BRCA1/2*-N: Normal breast tissue from *BRCA1* and *BRCA2* germline mutation carriers
CMI: Cumulative methylation index
CpG: Cytosine phosphate guanine
ER: Estrogen receptor
FFPE: Formalin-fixed paraffin-embedded
Non-*BRCA*-related-N: Normal breast tissue from patients not known to have a *BRCA1* or *BRCA2* germline mutation
HER2: Human epidermal growth factor receptor 2
MS-MLPA: Methylation-specific multiplex ligation-dependent probe amplification
PARP: Poly(adenosine diphosphate-ribose) polymerase
PR: Progesterone receptor
Sporadic-C: Sporadic breast carcinoma
TCGA: The Cancer Genome Atlas
